# Quantitative and qualitative assessment of structural magnetic resonance imaging data in a two-center study

**DOI:** 10.1186/1471-2342-12-27

**Published:** 2012-08-06

**Authors:** Sima Chalavi, Andrew Simmons, Hildebrand Dijkstra, Gareth J Barker, AAT Simone Reinders

**Affiliations:** 1 Department of Neuroscience, University Medical Center Groningen, and BCN Neuroimaging Center, University of Groningen, Groningen, The Netherlands; 2 King's College London, Institute of Psychiatry, Department of Psychosis Studies, London, UK; 3 King's College London, Institute of Psychiatry, Department of Neuroimaging, London, UK; 4 Biomedical Research Centre for Mental Health at South London and Maudsley NHS Foundation Trust and Institute of Psychiatry, King’s College London, London, UK; 5 Centre for Neurodegeneration Research, King’s College London, London, UK; 6 Department of Radiology, University Medical Center Groningen, University of Groningen, Groningen, The Netherlands

**Keywords:** Multi-center, Structural MRI, Freesurfer, SPM, Voxel based morphometry, Cortical thickness, Subcortical volumes, Reproducibility, Test-retest, Variability, Relative mean difference

## Abstract

**Background:**

Multi-center magnetic resonance imaging (MRI) studies present an opportunity to advance research by pooling data. However, brain measurements derived from MR-images are susceptible to differences in MR-sequence parameters. It is therefore necessary to determine whether there is an interaction between the sequence parameters and the effect of interest, and to minimise any such interaction by careful choice of acquisition parameters. As an exemplar of the issues involved in multi-center studies, we present data from a study in which we aimed to optimize a set of volumetric MRI-protocols to define a protocol giving data that are consistent and reproducible across two centers and over time.

**Methods:**

Optimization was achieved based on data quality and quantitative measures, in our case using FreeSurfer and Voxel Based Morphometry approaches. Our approach consisted of a series of five comparisons. Firstly, a single-center dataset was collected, using a range of candidate pulse-sequences and parameters chosen on the basis of previous literature. Based on initial results, a number of minor changes were implemented to optimize the pulse-sequences, and a second single-center dataset was collected. FreeSurfer data quality measures were compared between datasets in order to determine the best performing sequence(s), which were taken forward to the next stage of testing. We subsequently acquired short-term and long-term two-center reproducibility data, and quantitative measures were again assessed to determine the protocol with the highest reproducibility across centers. Effects of a scanner software and hardware upgrade on the reproducibility of the protocols at one of the centers were also evaluated.

**Results:**

Assessing the quality measures from the first two datasets allowed us to define artefact-free protocols, all with high image quality as assessed by FreeSurfer. Comparing the quantitative test and retest measures, we found high within-center reproducibility for all protocols, but lower *between-*center reproducibility for some protocols than others. The upgrade showed no important effects.

**Conclusions:**

We were able to determine (for the scanners used in this study) an optimised protocol, which gave the highest within- and between-center reproducibility of those assessed, and give details of this protocol here. More generally, we discuss some of the issues raised by multi-center studies and describe a methodical approach to take towards optimization and standardization, and recommend performing this kind of procedure to other investigators.

## Background

Assessment of brain tissue morphometry is becoming an important biomarker for diagnosis and treatment of a variety of neurological diseases [1-4]. High resolution structural magnetic resonance imaging (sMRI) facilitates quantitative insight into the normal human brain and to changes that occur due to pathology, e.g. in neuropsychiatric or neurological diseases. Changes in cortical thickness are manifested in normal aging [5] and with the progression of neuro-degenerative diseases such as Alzheimer’s disease [6,7], multiple sclerosis [8,9] and schizophrenia [10,11], while alterations in subcortical brain volumes have been reported in normal aging [12,13], Alzheimer's disease [14], Huntington's disease [15,16], and schizophrenia [10,17].

Recently, multi-center MRI studies have increased in popularity. They provide the opportunity to increase subject numbers by pooling data from different centers, which is particularly important for maximising recruitment rates and for studying rare diseases [18-21].

Although manually measuring the brain's volume (using manual tracing techniques) by experienced and trained tracers is often considered as a ‘gold standard’ [22,23], in large multi-center studies this approach becomes problematic because of the time required; automatic brain measurements are therefore often preferred. There are two types of measurements that can be made by automatic brain analysis tools: quantitative measures, which provide outputs such as brain volumetric and morphometric measures, intended for further analysis, and data quality measures, which give information about the quality of the images, intended to help assess the success of the data analysis approach. One important confound of combining images from different scanners and analysing them with automatic tools is that the volumetric and surface-based brain measurements derived from MR images can be dependent on the scanner manufacturer [24-28], field strength [24-26,28], MRI protocol [25,26,29,30], scanner drift over time [31] and data analysis tool employed [25,32,33], as well as the impact of a scanner upgrade [24,25]. Slight differences in these factors can have a considerable impact on the reliability and reproducibility of the results [20]. It is therefore important to determine whether there is an interaction between the acquisition protocol involved and the effect of interest, and to minimise any such interaction by careful choice of acquisition and analysis methods. Thus, the main aim of the present paper is to report a methodical approach to the choice of MRI protocol for multi-center studies, illustrating the approach using the results from a recent two center study. For our study, we optimized standard vendor derived brain structural MR protocols (with candidate sequences and parameters chosen on the basis of previous reports in the literature) based on their performance with respect to two commonly employed data analysis methodologies. We assessed suitability for the FreeSurfer surface- and volume-based image analysis tool and investigated the reproducibility within and between centers of quantitative measures extracted from FreeSurfer, along with measures from the SPM5 version of Voxel Based Morphometry [34-36].

In this study we collected datasets from two centers equipped with MR scanners with the same field strength and from the same manufacturer. By assessing summary measures of data quality provided by the FreeSurfer program from within-subject within-center datasets, we first aimed to determine the protocols with the highest data quality. Then, by assessing quantitative measures from both FreeSurfer and VBM, we aimed to assess the reproducibility of the MR images 1) within-center, 2) between-center and 3) after scanner upgrade. We report the protocol with the highest reproducibility, which is now in use in a study of Dissociative Identity Disorder (DID) (http://www.neuroimaging-did.com).

## Methods

### Data acquisition and subjects

In this two-center study, subjects were imaged at two different centers in the Netherlands (Groningen and Amsterdam) which were both equipped with Philips 3 T Intera MR scanners (Philips Medical Systems, Best, NL), referred to here as Center 1 and Center 2. Both centers used the manufacturer's standard 8-channel head coil and software version 2.6.3. We acquired and analyzed five datasets in order to evaluate the quality of images and the reproducibility of different MRI protocols within and between centers. All participants were healthy volunteers with no history of major psychiatric or neurological disease, and provided written informed consent for a study approved by the local ethics committee (Medisch Ethische Toetsingsingscommissie (METc) of the University Medical Center Groningen (UMCG) and Academisch Medisch Centrum (AMC)). For the time period of this study, Periodic Image Quality Tests (PIQT) data, which were collected by the manufacturer as a part of a standard Quality Assurance (QA) protocol, were obtained from both centers and the signal to noise ratio, artefact level and uniformity measurements were investigated.

### Comparing T1-weighted volume protocols

As a first step we recommend comparing a range of candidate T1-weighted volume protocols. In our case a single 27-year old healthy female was scanned using six candidate MRI protocols recommended by the vendor and MR physicists at the two centers. While FreeSurfer provided specific protocol recommendations for Siemens scanners, it is difficult to directly translate these to other manufacturer's platforms. We therefore investigated an MPRAGE (Magnetization Prepared RApid Gradient Echo) pulse sequence with similar parameters, along with a number of other pulse sequences and parameters capable of giving whole brain high resolution scans with isotropic or near isotropic voxels, in acceptable imaging times. The protocols included FFE (Fast Field Echo), equivalent to Fast Low Angle SHot (FLASH) on Siemens scanners, and 3D TFE (Turbo Field Echo), equivalent to MPRAGE on Siemens scanners, with 5 different voxel sizes (1.0, 1.05, 1.1, 1.2 and 2.0 mm), two phase encoding directions (right-left and anterior-posterior) and a variety of TR and TE settings (Table 1). This dataset was assessed using quality measures from FreeSurfer's cortical reconstruction process. As these measures are likely to be affected by small changes in the subject position between, or motion during, scans, a thorough visual inspection was made of all data, such that any datasets showing visible bulk motion or artefact likely to be subject-motion related could be excluded from further processing.

**Table 1:** Pulse sequence parameters used in the study

**Dataset**	**Protocol name**	**Protocol type**	**TR (ms)**	**TE (ms)**	**Flip angle**	**No. Slices**	**Scan time (sec)**	**in-plane resolution (mm2)**	**Phase encoding direction**	**Slice thickness (mm)**	**Slice gap (mm)**	**FC**^**a**^	**SB**^**D**^
Initial comparing T1-weighted protocols	A	FLASH	25	4.6	30	160	408	1.0x1.0	Right-left	2.0	−1	-	-
	B	MPRAGE	9.8	4.6	8	120	279	1.16x1.1	Right-left	1.2	0	-	-
	C	MPRAGE	7.6	3.5	8	160	614	1.0x1.0	Anterior-posterior	1.0	0	-	-
	D	MPRAGE	7.6	3.5	8	160	614	1.0x1.0	Anterior-posterior	1.05	0	-	-
	E	MPRAGE	9.8	4.6	8	120	279	1.16x1.1	Right-left	1.2	0	-	-
	F	MPRAGE	7.1	3.3	8	145	557	1.0x1.0	Anterior-posterior	1.1	0	-	-
Optimizing T1-weighted protocols	F	MPRAGE	7.1	3.3	8	145	557	1.0x1.0	Anterior-posterior	1.1	0	-	-
	F1	MPRAGE	7.1	3.3	8	145	557	1.0x1.0	Anterior-posterior	1.1	0	-	✓
	F2	MPRAGE	7.1	3.3	8	145	557	1.0x1.0	Right-left	1.1	0	-	-
	F3	MPRAGE	7.1	3.3	8	145	557	1.0x1.0	Right-left	1.1	0	-	✓
	C1	MPRAGE	9.5	5.3	8	160	614	1.0x1.0	Anterior-posterior	1.0	0	✓	-
	C2	MPRAGE	7.6	3.5	8	160	614	1.0x1.0	Anterior-posterior	1.0	0	-	✓
	C3	MPRAGE	10	5.6	8	160	614	1.0x1.0	Right-left	1.0	0	✓	-

FC^a^: Flow Compensation, manufacturer's default settings.

SB^D^: Saturation band, manufacturer's default settings.

### Improving T1-weighted volume protocols

The next step is to improve the quality of the images based on the results of the first stage comparison. The details of this stage may vary significantly depending on the results of the first stage. In our case, visual inspection of the images from the first comparison revealed a number of artefacts, in particular a pulsation artefact apparently arising from the carotids. Inconsistencies in phase and amplitude can lead to this kind of artefact, in which ghost images of the vessels or vessel walls are seen along the phase encoding direction. Pulsation artefact can be reduced by adding flow compensation which applies an additional gradient to eliminate phase differences for both stationary and moving spins at the echo time and/or by changing the orientation of phase encoding.. In this study seven variants of the most promising protocols used in the first step were tested (on the same 27-year old healthy female subject) in order to determine protocols which minimized these artefacts while retaining high quality as assessed by FreeSurfer's cortical reconstruction process. Parameter changes included changing the phase encoding direction, adding flow compensation or adding saturation bands using the manufacturer's default settings (Table 1). As previously, a thorough visual inspection was made of all data, such that any datasets showing visible bulk motion or artefact likely to be subject-motion related could excluded from further processing.

### Short-term two-center reproducibility

Good short-term multi-center reproducibility is a key minimum requirement for any longitudinal study. In our case the 27 year old healthy female subject was rescanned with two additional young healthy females (26 ± 1.73 years) at both centers with a one day interval, using the three best performing protocols in order to assess the reproducibility of the candidate protocols across centers. This dataset, which was acquired two weeks before a scanner upgrade at Center 1, was also used as part of a scanner upgrade assessment (see below).

### Long-term two-center reproducibility

In order to assess the long-term reproducibility of the protocols, the same three participants (27.83 ± 2.31 years) were re-scanned at both centers using the same three protocols 1.5 years later. Long-term reproducibility is also key to longitudinal studies, which normally take place over timescales of months to years. Scanner servicing, replacement of components and long-term scanner drift can all potentially impact on long-term reproducibility.

### Assessment of scanner upgrade

To investigate the effect of scanner upgrade the three participants were re-scanned one week after a scanner upgrade at Center 1, when the number of receive channels was changed from 8 to 32 and the scanner operating software was upgraded from 2.6.3 to 3.2.10, again using the same three candidate MR protocols. (Note that although this upgrade provided a 32 channel capability, for the current study the same 8-channel head coil was used before and after the upgrade). Generally it is best to avoid scanner upgrades part way through longitudinal studies, of course, though this will often not be possible due to other operational requirements.

### Data analysis

The main interest in our Dissociative Identity Disorder study is volumetric and cortical thickness comparison of different psychiatric groups, so in this study we concentrated on the results from two fully automated brain analysis segmentation tools: FreeSurfer version 4.5 (http://surfer.nmr.mgh.harvard.edu) and Voxel Based Morphometry (VBM) (as implemented within SPM5 (www.fil.ion.ucl.ac.uk/spm)). A brief description of segmentation procedures follows, with further details provided in previous publications [36,37].

The FreeSurfer processing pipeline includes both surface-based [38] and volume-based [37,39] streams. In brief, after preprocessing including motion correction, affine registration to Talairach space, bias field correction, intensity normalization and skull stripping, each voxel is classified as either white matter (WM) or non-WM based on intensity values and neighbor constraints. Hemispheres are separated from each other and an initial WM surface tessellation is generated for each hemisphere and smoothed on the basis of intensity gradients between WM and grey matter (GM) voxels. The white surface is then “nudged” to follow the intensity gradients between the gray matter and cerebrospinal fluid (CSF); this defines the pial surface, and cortical thickness is measured as the average of the shortest distance from the WM surface to the pial surface and from the pial surface to the WM surface [40]. For subcortical segmentation, FreeSurfer combines information about voxel intensity relative to a probability distribution for tissue classes with information about the spatial comparisons to neighboring voxel labels and spatial comparisons to a probabilistic training atlas; structures are determined by assigning each voxel to one of approximately 40 possible labels.

Structural images were also processed using VBM, implemented with Statistical Parametric Mapping software (SPM5) running under Matlab 7.0 (MathWorks, Natick, MA). First, structural images were segmented to determine GM and WM, and normalized to an asymmetric T1-weighted template in Montreal Neurological Institute (MNI) stereotactic space, in a recursive manner [36]. Then images were corrected for volume changes induced by spatial normalization (modulation) [35]. This “modulation” step involves multiplying the spatially normalized gray matter by its relative volume before and after spatial normalization. The resulting gray matter images were finally smoothed with an 8 mm isotropic Gaussian kernel (the default setting for VBM).

We performed two types of measurements:

i. Data quality measures: To determine the MR protocols with the highest performance, we extracted and analyzed summary measures of data quality (described below) from the FreeSurfer analysis of the first two stages and used these as proxies for general image quality.

ii. Quantitative volume and cortical thickness measures: In order to determine which protocols had the best reproducibility within and between centers, and also after scanner upgrade, we extracted quantitative measures (also described below) from FreeSurfer and VBM analyzed data from the short-term multi-center reproducibility, long-term multi-center reproducibility and scanner upgrade comparisons.

### Data quality measures

Artefacts can have an important negative impact both on the visually assessed quality of images, as well as on the performance of automated analysis techniques. Such artefacts include blurring and ghosting artefacts caused by motion or flow which can also influence the scan quality in subtle ways that can often only be assessed qualitatively. In this study all images were therefore visually inspected for artefacts, but in addition we also assessed “summary data quality” measures that, though not specific, are likely to be affected by many types of commonly occurring image artefacts. The FreeSurfer summary data quality measures we used are the “Euler number” and “Contrast to Noise ratio (CNR)” as suggested by Dr Bruce Fischl (http://www.mail-archive.com/freesurfer@nmr.mgh.harvard.edu/msg11456.html) and briefly described here. An examplar dataset from FreeSurfer (known as “Bert”), was also analyzed for comparison purposes. The summary data quality measures from each protocol were compared to the performance of the other protocols, and the protocols with the highest total score were selected for further investigation. In the current study we chose to weight all scores equally, but such a choice needs to be considered on a case by case basis, and for other studies it may be appropriate to give different weights to the factors.

#### Contrast-to-noise ratio (CNR)

CNR is the ratio of the difference in signal intensity between regions of different tissue types and background (noise) signal. The contrast must be sufficient to obtain robust brain measurements as many automated techniques rely on high contrast boundaries between brain and CSF, or between different tissues (e.g. GM and WM). Selecting an appropriate protocol and also increasing field strength can increase the CNR. CNR information is automatically computed by FreeSurfer [38]. For our purposes we assumed that the higher the CNR the higher the data quality, and the better the assumed performance for FreeSurfer and other segmentations techniques on such data.

#### Euler Number

It is very important that cortical reconstruction procedures can create a cortical surface model which is geometrically and topologically representative of the cerebral cortex. Lee et al. [41] note that “since the cerebral cortex has the topology of a 2-D sheet, a topologically correct surface model should have no holes and handles and the Euler number should be '2'.” For a surface which contains holes or handles, the Euler number is 2-2 g, where g is the number of defects. The Euler number is also automatically computed by the FreeSurfer pipeline [38], and we used this as a metric of cortical surface reconstruction quality: the higher the Euler number, the higher the data quality for FreeSurfer cortical reconstruction.

### Quantitative volume and cortical thickness measures

The quantitative volume and cortical thickness measures which were extracted in this paper are: cortical thickness and subcortical volume measurements from FreeSurfer, and total gray matter volume, total white matter volume and whole brain volume (WBV) from VBM-SPM5.

#### FreeSurfer quantitative measures

In our study, a *global* mean cortical thickness measurement for each subject was computed over the whole cortical surface, combining left and right hemispheres, while *regional* cortical thickness was assessed by limiting cortical thickness measures to one of five major cortical regions: frontal, parietal, temporal, occipital and cingulate.

Volumetric measurements were collected for six subcortical structures which are of particular interest in neuro-degenerative diseases - thalamus (T), caudate (C), putamen (PU), pallidum (PA), hippocampus (H) and amygdala (A). For each of these, the right and left hemisphere volumes were evaluated separately.

Depending on the nature of the multi-center study the most appropriate quantitative measures to assess will, of course, vary.

#### VBM-SPM quantitative measures

After the smoothing step in VBM, each voxel represents the local average amount of gray or white matter. In this study we report only the total WM and total GM volume results, as the CSF compartment is poorly defined by SPM5 and includes other tissue in non-negligible quantity (areas of skull bone and dura, scalp musculature, fat, upper cervical vertebrae, etc.). Whole brain volume (WBV) was defined in this case as the sum of WM and GM volumes.

### Statistical analysis

The reproducibility of each quantitative measure was assessed by calculating the relative mean difference (as a percentage) as described in equation (1) in which Q_tp1_ and Q_tp2_ indicate the quantitative measure extracted at time-point 1 and time-point 2, respectively. Reproducibility and relative mean difference are inversely related i.e. the lower the relative mean difference the higher the reproducibility and vice versa.

Relative mean difference (%) = 100*ABS(Q_tp1_ -Q_tp2_)/mean(Q_tp1_,Q_tp2_) (1)

Reproducibility of the protocols was then evaluated within-center, between-center and after scanner upgrade by calculating the relative mean difference in each of the following comparisons:

1) Within-center reproducibility: Center 1 test vs Center 1 retest and Center 2 test vs Center 2 retest.

2) Between-center reproducibility: Center 1 test vs Center 2 test and Center 1 retest vs Center 2 retest.

3) Post scanner upgrade reproducibility: Center 1 retest vs Center 1 upgrade.

## Results

### Data quality measures

#### Comparing T1-weighted volume protocols

Visual assessment revealed no datasets that needed to be excluded due to subject motion. Results of the initial FreeSurfer summary data quality measures of the comparing T1-weighted volume protocols (Table 1) are shown in Table 2. In this table each of the protocols was scored according to its rank relative to the other protocols.

**Table 2:** Freesurfer summary data quality measures of the initial T1-weighted volume protocols and their ranking relative to the other protocols in parentheses

		**Bert**	**A**	**B**	C*	**D**	**E**	F*
**Euler No.**	**Right**	−40	−396 (1)	−62 (5)	−66 (4)	−78 (3)	−94 (2)	−40 (6)
	**Left**	−58	−510 (1)	−88 (2)	−72 (4)	−62 (5)	−74 (3)	−52 (6)
**CNR**	**Grey/white**	2.02	1.50 (1)	2.21 (2)	2.5 (4)	2.59 (5)	2.26 (3)	2.86 (6)
	**Grey/CSF**	1.09	0.51 (1)	0.69 (2)	0.78 (5)	0.73 (3)	0.75 (4)	0.94 (6)
**sum of scores**			4	11	17	16	12	24

Protocols with better summary data quality measures are given higher scores.

Criteria*:*

Euler No : the less negative the better,

Grey/white CNR: the bigger the better,

Grey/CSF CNR : the bigger the better,

Total CNR : the bigger the better.

* The MRI protocols taken forward to the next step.

**Figure 1:**
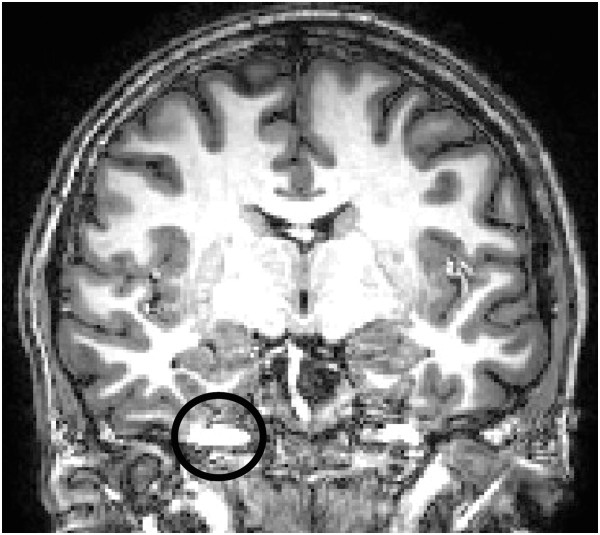
**Pulsation artefact detected in the acquired images of initial T1-weighted volume protocols dataset with selected protocols C and F.** Inconsistencies in phase and amplitude can result in this kind of artefact, which can be reduced by adding flow compensation and/or changing the phase encoding direction.

The results indicate that FLASH protocol (A) showed lower image quality than both the FreeSurfer example dataset (“*Bert”*) and all the other protocols, so this protocol was excluded from further analysis. Of the remaining protocols, the MPRAGE scans with the highest total score, i.e. protocols C and F were taken forward to the next stage. However, there were a number of artefacts present in the images acquired with these protocols, mostly pulsation artefacts as can be seen as ghost images of the vessels or vessel walls along the phase encoding direction in Figure 1, and as described above, a number of changes were therefore made to the protocols before the second dataset was collected.

#### Optimization of T1-weighted volume protocols

Table 3 shows the results of the summary data quality measure comparisons for seven protocols from the optimization step. Again, visual assessment revealed no datasets that needed to be excluded due to subject motion. All the protocols gave acceptable results, performing as well as or better than both the protocols in the initial comparisons (Table 2) and the FreeSurfer example dataset “*Bert*”. Visual inspection of images by two independent observers (SC and SR) suggested three artefact-free protocols (F2, F3 and C3), which were selected for further tests.

**Table 3:** Freesurfer data quality measures of optimized T1-weighted volume protocols

		**Bert**	**F**	**F1**	F2*	F3*	**C1**	**C2**	C3*
**Euler No**	**Right**	−40	−38	−32	−38	−34	−74	−52	−42
	**Left**	−58	−46	−42	−28	−44	−66	−72	−48
**CNR**	**Grey/white**	2.02	2.92	2.94	2.82	2.89	2.6	2.71	2.67
	**Grey/CSF**	1.09	1.004	1.004	0.99	1.024	0.92	0.84	0.99

* All the protocols show acceptable image quality results compare to the previous dataset and standard Freesurfer exemplar Bert. However, protocols F2, F3 and C3 were identified as artefact-free and selected for further tests.

### Quantitative volume and cortical thickness measures

No significant differences were found comparing the signal to noise ratio, artifact level and uniformity measurements of the PIQT data of the two centers (data not shown).

1) **Within-center reproducibility**

**Figure 2:**
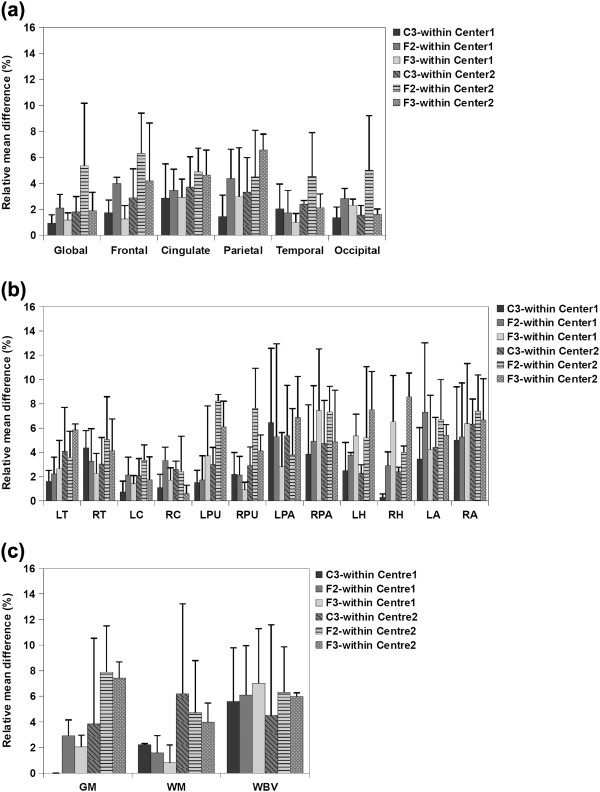
**Average relative mean difference (%) of a) cortical thickness, b) subcortical volumes and c) VBM measurements for Center 1 and Center 2 within-center comparisons.** Error bars show the standard deviation of the relative mean difference.

**Figure 3:**
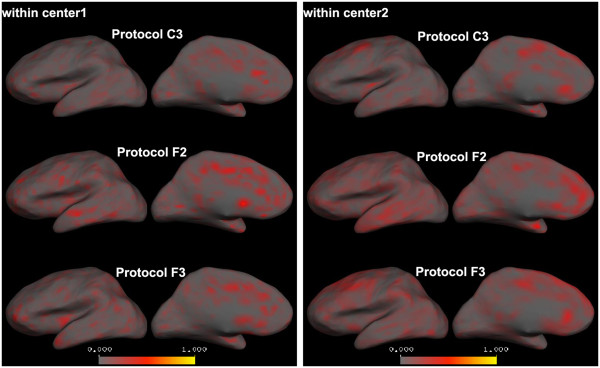
**Within-center average absolute cortical thickness differences calculated for each vertex on the left cortical surface (right hemisphere is similar).** Comparison between the two centers reveal that while all the protocols are highly reproducible in Center1, in Center2 only protocol C3 shows high reproducibility (less red areas) and the other two protocols show high cortical thickness differences especially in frontal and parietal regions.

a) Cortical thickness measurements

Figure 2(a) illustrates the average relative mean difference of the cortical thickness measurements from the within-center comparisons. As can be seen, all the protocols showed high reproducibility in Center 1, with low average relative mean difference (less than 5%) in all cases; However, the reproducibility (for Center 2) of protocols F2 and F3 was not as high as protocol C3 for global, frontal and parietal measurements. Voxel-wise maps of average absolute cortical thickness differences in Figure 3 show that Protocol C3 was highly reproducible at both centers, while protocols F2 and F3 showed lower reproducibility in the frontal and parietal regions, especially at Center 2.

b) Subcortical volume measurements

The average relative mean difference of the subcortical volumes shown in Figure 2(b) illustrate that for Center 1 the volumetric results of most of the structures were highly reproducible for all the protocols (relative mean difference less than 5%). Exceptions were the hippocampal volume acquired using protocols F3 and pallidum and amygdala volumes from all protocols (although the amygdala differences were lowest using protocol C3). However for Center 2, protocols F2 and F3 showed low reproducibility, especially for the putamen, pallidum, hippocampus and amygdala. Protocol C3 showed low reproducibility for pallidum and amygdala (relative mean difference greater than 5%), but again, amygdala differences were the lowest using protocol C3

c) VBM total volume measurements

Figure 2(c) shows the average relative mean difference of the VBM measurements (WM, GM, WBV) of the within-center comparisons. GM and WM measurements acquired using all the protocols were highly reproducible for Center 1 within-center scans, However, for Center2 within-center scans, average relative mean difference for GM measurements acquired using protocol F2 and F3, and WM measurements acquired using C3 and F2 were larger than 5%. Average relative mean difference for WBV measurements from both centers were in the same range for all protocols although protocol C3 was found to have the highest reproducibility for both centers.

2) **Between-center reproducibility**

**Figure 4:**
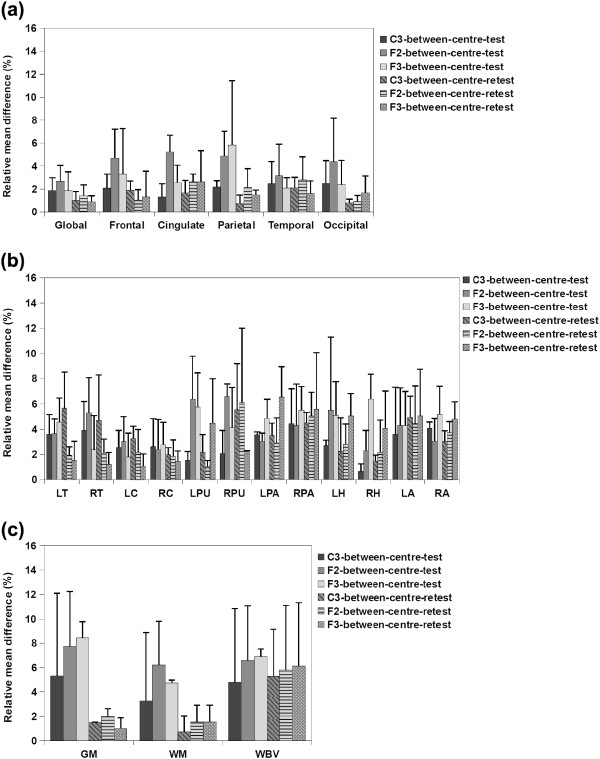
**Average relative mean difference (%) of a) cortical thickness, b) subcortical volumes and c) VBM measurements for between-center comparisons of the test and retest scans.** Error bars show the standard deviation of the relative mean difference.

**Figure 5:**
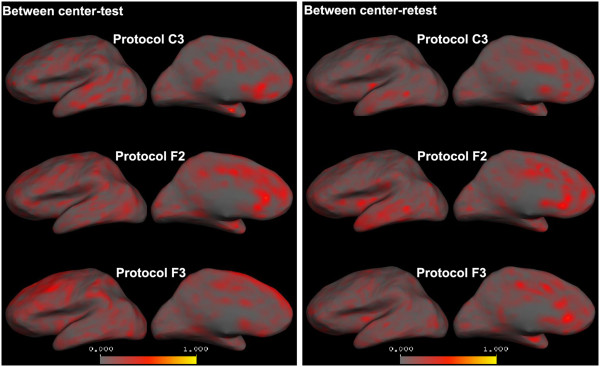
**Between-center average absolute cortical thickness differences calculated for each vertex on the left cortical surface (right hemisphere is similar).** While protocol C3 shows low average absolute global cortical thickness differences for both test and retest scans, protocol F2 shows high cortical thickness differences especially in frontal, parietal and cingulate regions of the test scans and protocol F3 reveals high cortical thickness differences in cingulate and parietal regions of the test scans.

a) Cortical thickness measurements

Figure 4(a) illustrates the average relative mean difference of between-center comparisons for the test and retest scans. As can be seen in this figure, for the baseline (test) scans, protocols F2 and F3 show lower reproducibility, especially in cingulate and parietal regions, in which they have average relative mean difference higher than 5%. Vertex-wise maps of average absolute cortical thickness differences (Figure 5) demonstrate that for both centers, while protocol C3 showed low global cortical thickness differences, protocol F2 and F3 showed high cortical thickness differences especially in the aforementioned regions. For the between center retest scans however all the protocols showed high reproducibility (average relative mean difference less than 5%).

b) Subcortical volume measurements

Average relative mean difference of subcortical volumes for the between-center studies shown in Figure 4(b) illustrate that for the baseline scans protocols F2 and F3 were not as reproducible as protocols C3, especially for putamen, pallidum, hippocampus and amygdala. For the retest scans, however, protocol C3 showed low reproducibility for thalamus, putamen and amygdala and protocols F2 and F3 showed low reproducibility for putamen, pallidum, hippocampus and amygdala, protocol F2 showed high average relative mean differences in the putamen, and protocol F3 showed low reproducibility in the pallidum, hippocampus and amygdala.

c) VBM volume measurements

Average relative mean difference of the VBM measurements for between-center comparisons, shown in Figure 4(c), demonstrates that for both baseline (test) and retest scans, protocol C3 is the highest reproducible protocol, with the average relative mean difference always less than 6% whereas protocols F2 and F3 showed higher average relative mean difference for GM and WBV measurements especially for the test comparisons.

3) **Post scanner upgrade reproducibility**

a) Cortical thickness measurements

**Figure 6:**
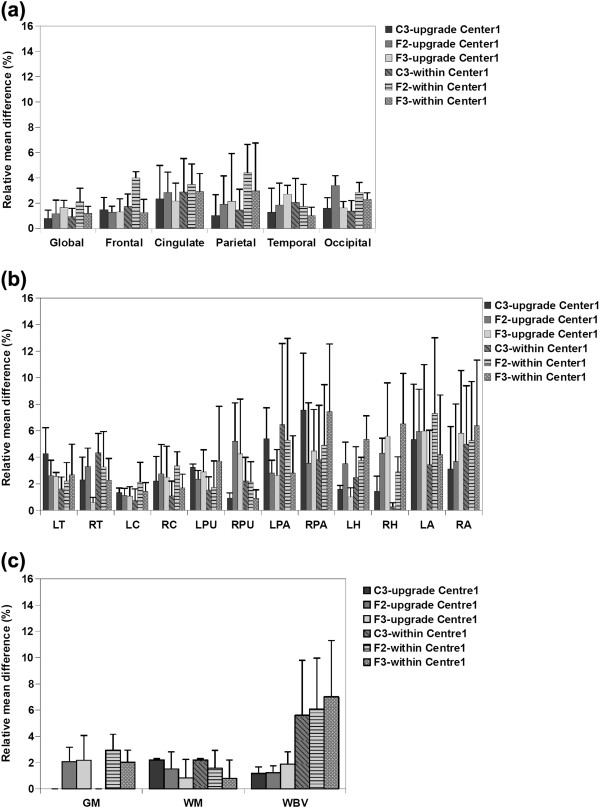
**Average relative mean difference (%) of a) cortical thickness, b) subcortical volumes and c) VBM measurements comparing Center1 images before and after the upgrade.** Error bars show the standard deviation of the relative mean difference. The average relative mean differences of the within Center1 measurements were also added for the comparison purposes.

**Figure 7:**
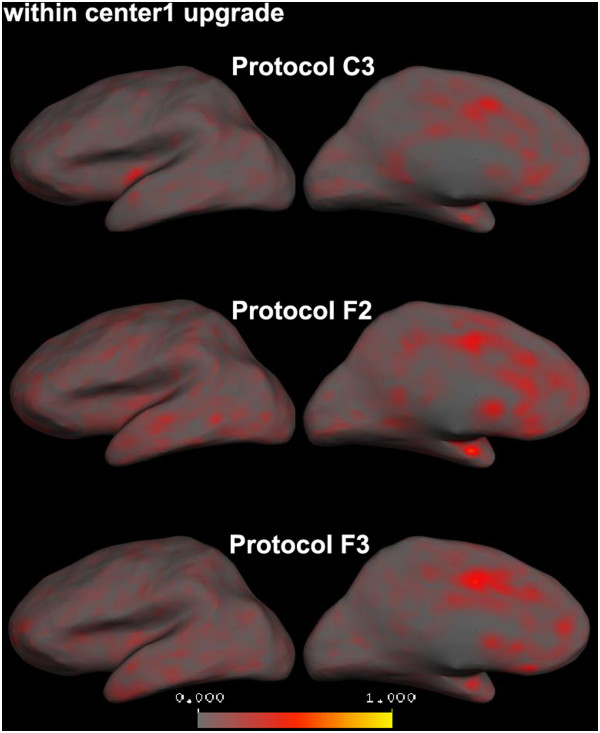
**Average absolute cortical thickness differences between before and after scanner upgrade images in Center1 calculated for each vertex on the left cortical surface (right hemisphere is similar).** All the protocols show low average absolute cortical thickness differences and therefore high reproducibility.

All the protocols showed low average relative mean difference measures (less than 5%) and therefore high reproducibility results comparing cortical thickness measurements from Center 1 data before and after the upgrade (Figure 6(a), within-center, Center 1 results were also added to this graph for comparison purposes). Voxel-wise average absolute cortical thickness differences illustrating this are shown (for the left hemisphere) in Figure 7.

b) Subcortical volume measurements

Average relative mean difference results of the subcortical volumes illustrated in Figure 6(b) show that the volumetric results of all the structures were highly reproducible using all the protocols except for the pallidum acquired using protocol C3 and putamen, hippocampus and amygdala acquired using protocols F2 and F3.

c) VBM total volume measurements

All the protocols showed low average relative mean difference (less than 5%) when the VBM total volume measurements before and after the scanner upgrade in Center 1 were compared (Figure 6(c)). The standard deviation of the average relative mean difference was also low.

## Discussion

Although combining structural MRI scans from different centers provides an opportunity to increase the statistical power of brain morphometric analyses in neurological and neuropsychiatric disorders, one important confound is the potential for scanner and MRI protocol effects to introduce systematic errors, thus making the interpretation of results difficult. In this study, we describe a methodical approach to choosing a T1-weighted volume, based on five steps. We demonstrated our approach for a two-center study, but believe that it can be generalized to larger multi-center studies. We acquired five datasets at two different centers (equipped with scanners from the same manufacturer with the same field strength): two within-subject within-center datasets for an initial comparison of T1-weighted volumes and subsequent optimization of the best performing of these protocols, two between-subject between-center datasets (short-term and long-term comparisons), and one between-subject, within-center dataset after a scanner upgrade in one of the centers. By analysing the summary data quality and quantitative measures extracted from FreeSurfer and VBM (as implemented within SPM5) we aimed to determine an optimised MRI protocol which gave high contrast to noise ratio/image quality (as evidenced by FreeSurfer measures), had minimal image artefacts and was reproducible across centers and over time.

For our initial assessment of image quality, after visual inspection for artefacts we extracted and examined the data quality measures (Euler number and contrast to noise ratio) and used these to compare the scans within the initial T1-weighted volume comparison with each other and with the examplar dataset from FreeSurfer. These quality measures were suggested by Dr. Fischl (http://www.mail-archive.com/freesurfer@nmr.mgh.harvard.edu/msg11456.html) and, while they are inherently inter-related (as the quality of the segmentation of the cortical surface depends in contrast and contrast to noise) it is important that measures are included that assess the “down stream” effects of image quality on image analysis as well as simple acquisition-related measures. The results indicated that the performance of FreeSurfer for reconstructing the surfaces was higher for MPRAGE sequences than the FLASH sequences. This is consistent with the results of Tardif et al. [42] and Deichmann et al. [43] that showed that because of their higher CNR images, MPRAGE sequences improve the accuracy of tissue classification. Therefore MPRAGE sequences are better options for segmentation tools such as FreeSurfer and VBM. Consequently, the FLASH sequence (protocol A) was rejected from further analysis, and from the remaining MPRAGE sequences we selected the two with the highest total summary data quality ranks (protocols C and F).

The top performing T1-weighted volumes were optimized in order to generate artefact-free protocols while retaining high image quality as assessed by FreeSurfer. We did this by applying a combination of changes in the parameter settings of the protocols including changing the phase encoding direction and/or adding flow compensation and/or saturation band. The extracted data quality measures indicated that image quality remained high enough (compared to “*Bert*” dataset and to the initial T1-weighted protocols) for all the 7 protocols in this dataset and we therefore visually inspected the images in order to select the best three artefact-free protocols to take forward for further testing. This dataset demonstrated that relatively minor changes to the protocol could have measurable effects on overall data quality, and emphasised the necessity for optimisation of protocols for the particular analysis to be performed.

Within-center comparisons of the quantitative measures from the short-term two-center and long-term two-center datasets with a scan interval of 1.5 year (Figure 2 and 3) indicated that for Center 2 cortical thickness comparisons, protocols F2 (with right-left phase encoding direction) and F3 (with right-left phase encoding direction and saturation band) showed low reproducibility in frontal, cingulate and parietal regions whereas all the regions were highly reproducible when using protocol C3 (with right-left phase encoding direction and flow compensation). In Center 1, on the other hand, all the protocols showed a high reproducibility for within-center cortical thickness comparisons. Within-center comparisons of a selection of subcortical structure volumes also indicated differences in the performance of the protocols, with protocol C3 showing higher reproducibility in most regions for measurements from both centers. VBM total volume results also showed lower reproducibility for GM measurements acquired using F2 and F3 than C3 and WM measurements acquired using C3 than F2 and F3. Whole brain volume measurements found to be more reproducible using C3 than the other protocols for both centers. These findings not only suggest that, for this study, protocol C3 gave the highest reproducible with respect to quantitative measures after a 1.5 year scan interval, but also highlights the importance of evaluating reproducibility at all sites in multi-center studies. While the Center 2 and Center 1 scanners are nominally identical, performance varied between these sites. Takao et al. [31] have reported similar findings, showing that even with scanners of exactly the same model (3 T General Electric scanners in their case) scanner drift and inter-scanner variability could cancel out the effects of genuine longitudinal brain volume changes. Since the PIQT data of the QA protocol did not show any significant difference between the two centers, the fact that all the protocols performed better at Center1 than at Center2 might reflect a subtle bias due to the fact that the optimization steps were performed for Center1 and the protocols then simply transferred to the other site. In this study, because of considerations of cost and time we chose to perform the initial comparison of T1-weighted volumes at one center and to use the best performing protocols from this center at both centers. However the ideal procedure for a multicenter study would be to: 1) perform and compare the QA information from the different centers, 2) acquire the first two steps at all sites and chose the best performing artefact-free protocols at all centers and then 3) perform the reproducibility tests. Furthermore, within-center variations were found comparing test and retest measurements at each of the centers which were highest for some of the protocols. One of the goals of the present study was thus to highlight the importance of reproducibility studies such as these, since this kind of within-center variation is not predictable a-priori. Han et al. [24] and Jovicich et al. [25] reported similar small variations in cortical and subcortical measurements, in their case comparing the scans from two different sessions with a 2-week scan interval (short-term scan interval). In the current study we compared the measurements after a long-term scan interval and showed that although some of the measurements showed lower reproducibility, it was possible to find a protocol and set of scan parameters that gave reproducible measures even over this longer interval.

Between-center comparison of cortical thickness from the short-term two-center comparison indicated that, with protocols F2 and F3 the reproducibility was lower for frontal, cingulate and parietal regions. However, with these two protocols, the differences in these regions were less in the long-term two-center retest comparisons. The reproducibility of all the regions in both the between-center test and in the between-center retest was higher using protocol C3. In the subcortical comparisons, the reproducibility of several volumes were low for protocols F2 and F3 in the initial between-center test scans while protocol C3 showed highly reproducible results. For the between-center retest scans also low reproducibility was found in several volumes using protocols F2 and F3 and protocol C3 found to be the most reproducible one. With respect to VBM total volume measurements, GM and WM measurements of the retest scans acquired using all the protocols were highly reproducible and for the GM and WM of the test scans and also WBV of both test and retest scans, protocol C3 found to be the most reproducible protocol. Again, these findings are important since they indicate that although both centers were equipped with the scanners of the same field strength and from the same manufacturer, the reproducibility was not always high and can be improved by carefully selecting the acquisition protocol.

The reproducibility of the quantitative measures was also examined after a scanner upgrade at Center 1. The reproducibility of all the protocols was relatively high for all the quantitative measures, even in problematic regions and structures, for both the within-center and between-center comparisons. These results are in line with reports from Han et al. [24], Stonnington et al. [44] and Jovicich et al. [25] in which the morphometric brain measurements did not significantly vary after even major scanner upgrades. Our results of between-center comparisons also indicate that the cortical thickness and VBM measurements difference across the centers were reduced after scanner upgrade at Center1. We speculate that this may be because of general servicing and tuning of the scanner during the upgrade but were not able to specifically assess this.

Ideally in large-scale multi-center and longitudinal studies which involve scanning very large number of subjects in several different centers, such as the Alzheimer's Disease Neuroimaging Initiative (ADNI) study [13] and Schizophrenia Twin and Relatives (STAR) study [45], using several human subjects for calibration procedure is preferred to get more precise results. In relatively small studies like ours (http://www.neuroimaging-did.com), however, which only involves scanning 50 subjects in each of the two centers, scanning a large number of calibration subjects is not feasible in terms of cost or time. Therefore, we decided to perform the calibration study using a small number of volunteers which is a potential limitation of this study. An additional potential confound is the impact of subject motion on the Euler number and other assessment measures; in the current study we control this by visual assessment of scans, but with a larger number of calibration subjects and scans, more quantitative methods could be applied to assess the effects.

Another important issue to consider is that of longitudinal studies. As was shown in the current study, within-center long-term reproducibility precision may vary to some extent depending on the MR protocol and these variations could be confounding factors. Systematic differences between scans acquired at different times could be mis-interpreted as real brain volumetric changes. Therefore, longitudinal studies need to consider performing reproducibility tests with a larger sample size and on a regular basis, and need to be appropriately powered. They also need more robust techniques than cross sectional measurements, which is particularly important when more than one center is involved in a longitudinal study. The results of such studies may also allow inter-site differences in accuracy to be assessed and if necessary allowed for by calibrations [45].

To summarize, our results showed that while several of the protocols showed promise, with high FreeSurfer performance/image quality and being artefact-free, one of the protocols (C3) gave the highest reproducibility. Determining this a-priori would have been impossible without acquiring and assessing these datasets. Since we scanned only three participants in the current study, we chose not to statistically compare the quantitative measures, but were nevertheless able to draw useful conclusions from the results.

The approach described here could be applied to protocol optimization across centers for other multi-center studies. These findings suggest researchers planning to perform multicenter studies should consider performing assessments such as these to ensure that by pooling data from different centers they are not unnecessarily reducing the power of their study due to variance from unexpected inter- or intra-site differences.

## Conclusions

In conclusion, in this study assessing the summary data quality measures helped us to find the protocols with the best reconstructed surface and the highest contrast-to-noise ratio (important for brain morphometric analysis, especially when using FreeSurfer). Evaluating the quantitative measures assisted us to specify the protocol with the highest reproducibility both for within- and between-center comparisons, which is crucial not only in multi-center but also in longitudinal studies. We strongly recommend assessing both quantitative and summary quality measures within and across the centers for multi-center studies, in order to ensure optimal behaviour of FreeSurfer, VBM and other similar methodologies, and to therefore enhance the trustworthiness of the final results.

## Abbreviations

MRI, Magnetic Resonance Imaging; sMRI, structural MRI; VBM, Voxel Based Morphometry; MPRAGE, Magnetization Prepared Rapid Gradient Echo; FLASH, Fast low Angle Shot; GM, Gray Matter; WM, White Matter; CSF, Cerebrospinal Fluid; WBV, Whole Brian Volume; CNR, Contrast to Noise Ratio; T, Thalamus; C, Caudate; PU, Putamen; PA, Pallidum; H, hippocampus; A, amygdala.

## Competing interests

The authors declare that they have no competing interests.

## Authors' contributions

All the co-authors have made substantial contributions to one or more of the following: the conception and design of the study; acquisition of data; analysis and interpretation of data. All have also been involved in drafting and revising the article, and have approved the final version to be published.

## Pre-publication history

The pre-publication history for this paper can be accessed here:

http://www.biomedcentral.com/1471-2342/12/27/prepub
